# Oxidative damage and inflammation in obese diabetic Emirati subjects supplemented with antioxidants and B-vitamins: a randomized placebo-controlled trail

**DOI:** 10.1186/1743-7075-10-21

**Published:** 2013-02-04

**Authors:** Salah Gariballa, Bachar Afandi, Mamoun AbuHaltem, Javed Yassin, Hosam Habib, Wissam Ibrahim

**Affiliations:** 1 Internal Medicine, Faculty of Medicine & Health Sciences, Al Ain, UAE; 2 Tawam hospital, Al Ain, UAE; 3 Nutrition & Health, Faculty of Food and Agriculture, United Arab Emirates University (UAEU), Al Ain, UAE

**Keywords:** Diabetes, Obesity, Inflammation, Antioxidants, Oxidative damage

## Abstract

**Background:**

Obesity and related morbidities are reaching epidemic proportions in the Arab populations. Possible mechanisms that link obesity/visceral fat to diabetes and cardiovascular (CVD) complications include inflammation and increased oxidative stress. The aim of this study is to test whether supplementary antioxidants with B-group vitamins enhance antioxidant capacity and/or mitigate oxidative damage and subclinical inflammation in obese diabetic patients.

**Methods:**

Hundred diabetic patients were randomly assigned to receive either oral dose of daily B-group vitamins (1.67 mg folic acid, 1.67 mg vitamin B-2, 20 mg vitamin B-6, 0.134 mg vitamin B-12) and antioxidant vitamins (221 mg of α-tocopherol and 167 mg of vitamin C) [n = 50], or an identical placebo [n = 50] daily for 90 days. Blood was obtained before treatment, and after 90 days for measurements of plasma antioxidant vitamins status, markers of oxidative damage [malondialdehyde (MDA) and protein carbonyls] and inflammation (C-Reactive Proteins [CRP], IL6 & TNFα).

**Results:**

*Supplementation* with antioxidant and B-group vitamins increased plasma concentration of vitamin E and folate and reduced homocysteine in the intervention groups compared with the placebo group. Vitamin B12 improved in the supplement group compared with the decline seen in the placebo group however, this did not reach statistical significance. Vitamin C declined in both groups but more so in the intervention group. Both MDA and Protein carbonyls increased in both the supplement and the placebo group. IL6 concentration increased in both groups but less so in the supplement group (p = 0.023). TNF showed more pronounced decline in the supplement group compared with the placebo group but the difference between cumulative changes did not reach statistical significance (p = 0.204). CRP concentrations declined in the supplement group in contrast to the rise seen in the placebo group however, the difference between cumulative changes was not statistically significant (p = 0.205).

**Conclusions:**

*Antioxidants* supplementation with B-group vitamins enhances antioxidant capacity, and may have an anti-inflammatory effect in obese diabetic patients.

## Introduction

The Middle Eastern countries including the United Arab Emirates (UAE) have been through rapid socioeconomic and social changes with urbanization over the last 40 years. Accompanying changes in diet and lifestyle are therefore leading to growing epidemic of overweight/obesity, type 2 diabetes mellitus and other related cardiovascular disease (CVD) [1]. People with Obesity and type 2 diabetes mellitus (DM) have increased risk of death from CVD. Possible mechanisms that relate obesity and diabetes to increased CVD risk include inflammation, oxidative damage, and related insulin resistance. In obese patients subclinical inflammation has been found to correlate with markers of oxidative stress in adipose tissue and this may be the mechanism for obesity-related metabolic syndrome, insulin resistance and diabetes mellitus. Furthermore both oxidative stress and low-grade inflammation may be causatively linked to the development, progression and complications of diabetes in obese patients [2]. There is clear evidence that oxidative stress and sub-clinical inflammation play a role in the development of CVD [3]. Furthermore emerging experimental and clinical evidence is supporting the presence of increased oxidative stress and inflammation in patients with obesity and type 2 diabetes. Factors that reduce oxidative stress and attenuate inflammation could provide an important tool to reduce the burden associated with obesity and related chronic disease including diabetes and CVD. Dietary supplements with antioxidant and related anti-inflammatory effects may present a novel strategy of controlling and reducing complication of obesity at the population level [4,5].

B-group vitamins are also known to have homocysteine-lowering effects, which is relevant because homocysteine does increase oxidative damage and has been implicated as a risk factor for CVD [6]. In addition to its homocysteine-lowering effects folate has also been ascribed antioxidant properties. The potential synergistic interactions between conventional antioxidants and B-group vitamins have not been studied in obese diabetic patients. Furthermore we have previously shown that B-group vitamins have antioxidant effects independent of their homocysteine-lowering effects and that antioxidant with B-group vitamin supplement in CVD patients significantly reduced markers of inflammation [7].

Although the UAE has a high prevalence of obesity and related diabetes mellitus at present, very little is known about the factors affecting obesity and associated morbidities including diabetes and CVD in the UAE [1]. The aim of this study was, therefore, to test the effect of antioxidants and B-group vitamins supplement on antioxidant capacity and markers of oxidative damage, and inflammation in obese diabetic patients.

## Material and methods

All patients with type 2 diabetes mellitus visiting the diabetes centre at the Tawam hospital for regular follow up of their diabetes were considered for the study. Tawam hospital is one of the two main teaching hospitals in the city of Al Ain serving a total population of 400,000. Patients aged 18 years and above with type 2 diabetes mellitus were approached and invited to take part in the study. Individuals with severe chronic clinical or psychiatric disease, participating in other intervention trials, on dietary supplements and those unable to give an informed written consent were excluded. The Local research ethical committee has approved the study.

Following informed written consent and their recruitment to the study, eligible patients had a fasting 10 ml of blood taken for measurements of antioxidants, B group vitamins, and related nutritional and biochemical variables at baseline. Patients were then randomly assigned to receive a capsule of antioxidant vitamins [221 mg of α-tocopherol and 167 mg of vitamin C] and B-group vitamins [1.67 mg folic acid, 1.67 mg vitamin B-2, 20 mg vitamin B-6, 0.134 mg vitamin B-12]; or an identical placebo daily for 90 days. Patients otherwise managed according to standard practice. Clinical assessment that included control of diabetes and associated risk factors and complications was also performed at baseline, and repeated at 3 months.

### Supplements and placebo

The Placebo tablet was identical to the active supplement vitamin capsule. No physician, investigator, nurse or a patient could differentiate between the active treatment capsule and the placebo capsule.

#### Compliance with trial medications

The compliance of the volunteers randomized to the supplements and placebo was assessed by: Counting the remaining supplements tablets at follow up visits and analysis of blood vitamin levels collected immediately after the end of the supplement period.

#### Measurements

Anthropometric data including body weight, height fat mass, fat-free mass were measured using Tanita body composition analyser. Waist circumference was measured using a flexible plastic tape.

### Blood samples

Fasting blood samples were drawn into 2 vacutainer tubes, containing potassium EDTA as anticoagulant. The samples were thoroughly mixed at room temperature and immediately transferred to the laboratory. Both tubes were centrifuged immediately for 10 min at 4000 rotations/min. Plasma and serum were collected and stored at −80°C for future determinations of biochemical outcome measures. *Antioxidants:* Vitamins C, E, and A, analysis were carried out using HPLC with fluorescence and ultraviolet detectors. This was performed on a Waters (Milford, MA) system gradient liquid chromatography (model515) with auto injector (model 717). We used commercially available enzyme-linked immunosorbent assay (ELISA) methods to measure plasma TNF and IL6. CRP was measured by standard methods using Synchron Clinical System (UniCel DxC-800) from Beckman Coulter, Inc., Fullerton, CA, USA. *Lipid Peroxidation:* The concentration of the lipid peroxidation product MDA was measured in the serum by the modified procedure of Li and Chow [8]. *Protein Oxidation:* The content of protein-bound carbonyls used to assess the extent of protein oxidation, was determined spectrophotometrically at 530 nm by the 2, 4-dinitrophenylhydrazine method of Levine et al. [9]. For vitamin B12 and Folate analyses, we used reagent kit from access reagent kit from BACKMAN COULTER. Plasma homocysteine was measured by Fluorescence Polarization Immunoassay (FPIA) technology using reagent kit (5 F51-20) for AxSYM system from Abbott Laboratories, IL, USA. We analyze all these samples on unicel DXI 800 access immunoassay system and unicel DXC 880i synchrone access clinical system. The local Pathology Laboratory performed other routine tests including full blood count, serum lipids, glucose, HbAic, albumin, BUN and LFTs.

### Statistics and analysis

#### Randomization strategy

The randomization sequence was generated using computer generated tables, concealed in sequentially numbered sealed opaque envelopes and kept in a clerical office.

#### Sample size calculation

A previous study has shown that supplementation of diet with 400 mg of vitamin C increased plasma vitamin C by 45% [10]. Therefore a sample size of 96 diabetic patients (48 treatment and 48 controls) would allow the detection of a 30% difference between groups in total plasma vitamin C concentrations with 80% power and type 1 error probability of < 0.05.

### Analysis

Regression analysis was used to test for treatment effect after adjusting for imbalance in baseline scores and log transformation for skewed data [11]. p value < 0.05 was considered significant. Spearman rank correlation and Mann-u-Whitney test were also used to test for correlations and differences in baseline scores respectively.

#### Quality assurance

To optimize accuracy of data collection, entry and analysis, two individuals performed quality assurance by independently checking the accuracy of data entry performed. A random audit of 20 cases of the entered data against the paper based forms was done twice by two different operators, and found that, more than 96% of the data entry process was accurate.

## Results

**Figure 1:**
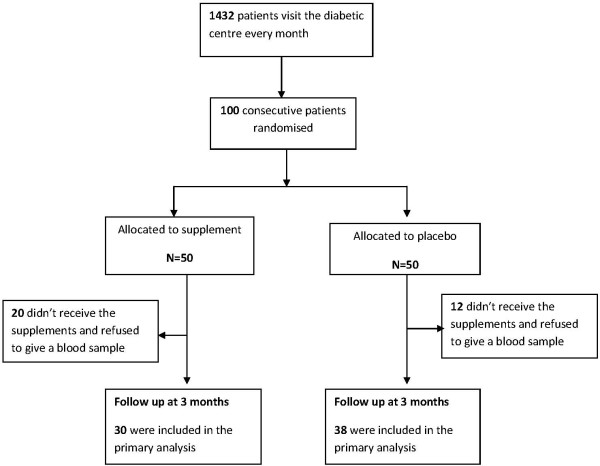
Enrolment, treatment and follow up of study patients.

Hundred diabetic patients were recruited to the study. Thirty-eight of those who received the placebo and 30 of those who received the supplements over three month’s period came for 3 months follow up and agreed to give a blood sample. Exclusions were due to refusal to give a blood sample at the follow up appointment. The Figure 1 details the recruitment and intervention process and 3-month follow up. Table 1 shows baseline characteristics of the treatment and placebo group. The 2 groups were comparable on entry into the study with respect to age, adiposity, duration and treatment of diabetes and other clinical and biomedical measures. Although there were some differences in some baseline characteristics such as gender, smoking, previous hypertension and total cholesterol level, these differences did not reach statistical significance.

**Table 1:** Baseline characteristics placebo and supplement group (Median Q1-Q3, unless stated otherwise)

		**Placebo**	**Supplement**	**P value**
		**(n = 50)**	**(n = 50)**	
**Age**		51 (42–60)	52 (44–56)	0.964
**Male: female** (n)		18:32	23:27	0.312
**Smoking** (n)		2	6	
**Duration of diabetes** (yrs)		2.0 (1–3)	3 (1–3)	0.258
**Previous Hypertension** (n)		34	28	0.219
**Previous IHD** (n)		8	7	0.781
**High cholesterol** (n)		36	38	0.650
**Number of drugs/patient**		2.2	2.3	0.849
**Treatment of diabetes** (n)	**Diet**	2	2	0.059
	**Tablet**	36	28	
	**Insulin**	9	18	
	**Both**	3	2	
**Other treatments,** n (%)				
ACE inhibitors		13 (26)	12 (24)	0.863
Calcium channel blockers		1 (2)	2 (4)	0.548
B blockers		14 (28)	16 (32)	0.616
aspirin		36 (72)	40 (80)	0.259
Statins		38 (76)	36 (72)	0.773
**Body mass index**		32 (29–36)	31. (26–35))	0.278
**Fat mass** (kg)		36 (28–43)	35 (29–43)	0.683
**Waist circumference** (cm)		104 (95–115)	100 (95–110)	0.281
**Systolic BP** (mmHg)		135 (121–145)	136 (120–153)	0.490
**Diastolic BP** (mmHg)		79 (70–85)	80 (73–86)	0.304
**HbAic** (%)		7.8 (7.0-9.3)	7.7 (6.4-9.0)	0.780
**Total cholesterol** (mmol/L)		4.1 (4.0-5.0)	4.3 (3.8-4.9)	0.402
**Triglycerides** (mmol/L)		1.0 (0.83-1.44)	1.35 (0.75-1.7)	0.335

Ninety diabetic patients out of 100 were either overweight (BMI 25–29.9), [n = 30] or obese (BMI ≥30), [n = 60].

### Supplementation

*The median (Q1-Q3) number of tablets (maximum 90 tablets) taken by the supplement and placebo group were 90 (70–90) and 85 (70–90) tablets respectively.* Vitamin supplementation increased plasma vitamin E and serum folate and reduced total plasma homocysteine levels in the intervention groups compared with the placebo group, (Tables 2). Vitamin B12 improved in the supplement group compared with the decline seen in the placebo group however, this did not reach statistical significance. Vitamin C declined in both groups but more so in the intervention group. Both MDA and Protein carbonyls increased in the supplement and the placebo group [p = 0.013 and 0.000 respectively (Table 2)]. MDA showed significant correlation with triglycerides but not other lipid parameters at 3 months (r = 0.47, p =0.001). Changes in vitamin A and GSH were not statistically significant. Table 3 shows the inflammatory markers profile at baseline and after 3 months of supplements. IL6 concentration increased in both groups but significantly less so in the supplement group (p = 0.023). TNF showed more pronounced decline in the supplement group compared with the placebo group but the difference between cumulative changes did not reach statistical significance (p = 0.204). CRP concentrations declined in the supplement group in contrast to the rise seen in the placebo group however the difference between cumulative changes was not statistically significant (p = 0.205).

**Table 2:** Baseline and 3 months plasma antioxidants and markers of oxidative damage in the intervention group compared with the placebo group (mean SD)

		**Placebo**	**Supplement**	**B (95% C.I) +**	P value*
		**(n = 50)**	**(n = 50)**		
**Vitamin C** (mg/l)	Baseline	23.8 (16.8)	33.00 (20.1)		
	3 months	19.5 (12.1)	18.9 (12.8)	−0.3 (−6.5, 5.8)	0.913
**Vitamin E** (mg/l)	baseline	7.3 (4.5)	8.6 (3.2)		
	3 months	7.6 (4.6)	11.4 (4.5)	3.1 (0.9, 5.3)	0.006
**Vitamin A** (mg/l)	Baseline	0.578 (0.29)	0.797 (0.34)		
	3 months	0.624 (0.39)	0.844 (0.29)	0.1(−0.12, 0.22)	0.541
**Glutathione**(nM/ml)	Baseline	0.231 (0.27)	0.245 (0.31)		
	3 months	0.139 (0.13)	0.169 (0.15)	0.03 (−0.04, 0.10)	0.346
**Folate** (nmol/l)	baseline	18.2 (8.9)	18.95 ((8.1)		
	3 months	18.7 (8.6)	32.4 (11.9)	13.5 (9.7, 17.4)	0.001
**B12** (pmol/l)	Baseline	236 (103)	179 (93)		
	3 months	227 (99)	252 (191)	93 (48, 139)	0.001
**Homocysteine**	Baseline	10.3 (3.2)	12.7 (4.5)		
(mmol/l)	3 months	10.7 (3.3)	11.5 (3.3)	−0.33 (−1.8, 1.2)	0.657
**Protein carbonyl**	baseline	0.797 (0.28)	0.456 (0.46)		
(nmol/mg)	3 months	1.080 (0.46)	0.737 (0.35)	−0.34 (−0.56, -0.13	0.002
**MDA** (nM/ml)	Baseline	16.7 (15.8)	6.1 (8.9)		
	3 months	18.5 (9.9)	19.7 (11.2)	−0.64 (−5.96, 4.67)	0.810

*3 months values were regressed on baseline values and intervention (placebo 1/supplement 2).

**+**B = regression coefficient (95% confidence interval).

**Table 3:** Baseline and 3 months inflammatory markers in the intervention group compared with the placebo group (mean SD)

		**Placebo**	**Supplement**	**B (95% C.I) +**	P value*
		**(n = 50)**	**(n = 50)**		
**IL6** (pg/ml)	Baseline	3.42 (2.22)	2.49 (1.32)		
	3 months	5.40 (2.53)	3.35 (1.99)	−1.25 (−2.33, -0.18)	0.023
**TNFα** (pg/ml)	baseline	1.26 (1.63)	1.66 (2.24)		
	3 months	1.15 (0.8)	0.96 (0.21)	−0.22 (−0.56, 0.12)	0.204
**CRP** (mg/l)	Baseline	11.6 (8.9)	10.1 (8.6)		
	3 months	15.1 (15.9)	8.4 (3.4)	−2.5 (−6.3, 1.4)	0.205

*3 months values were regressed on baseline values and intervention (placebo 1/supplement 2).

**+**B = regression coefficient (95% confidence interval).

## Discussion

The main findings of this study were that supplementary antioxidants with B-group vitamins enhanced individual antioxidant capacity and mitigated inflammation in obese diabetic patients. We found no significant effects of antioxidants and B-group vitamins together on markers of oxidative damage possibly due to the lower dose of the vitamins used or small sample size or a combination of both.

Possible mechanisms that relate obesity and diabetes to increased CVD risk include inflammation, oxidative damage, and related insulin resistance. In obese patients subclinical inflammation has been found to correlate with markers of oxidative stress in adipose tissue and this may be the mechanism for obesity-related metabolic syndrome, insulin resistance and diabetes mellitus. Furthermore both oxidative stress and low-grade inflammation may be causatively linked to the development, progression and complications of diabetes in obese patients [2-5]. Oxidative stress is defined as imbalance between the generation of free oxygen radicals and the antioxidant defense system and results from increased production of reactive oxygen species known to trigger cytotoxic reactions that are damaging to membrane lipids, proteins, nucleic acids and carbohydrates. A number of studies revealed the link between oxidative stress, obesity, diabetes and other related complications. Recent research does indeed support a close link between oxidative stress and diabetes evolution, revealing that oxidative stress occurs before the appearance of clinical manifestations of late diabetic complications, suggesting a key role in the pathogenesis of the disease [2]. Results from our study clearly show increased oxidative stress in diabetic subjects with time, as evidenced by a significant increase in MDA and protein carbonyls and reduction of vitamin C and GSH in both the placebo group and the intervention group. In addition a number of studies have reported an association between oxidative stress and insulin resistance and that some antioxidants may improve insulin resistance [4,5]. A recent systematic review and meta-analysis has reported that increasing daily intake of green leafy vegetables could significantly reduce the risk of type 2 diabetes and this should investigated further [12]. Another recent data also suggest that dietary antioxidants intake may be a predictor of the risk to develop metabolic syndrome features such as adiposity or impairments in systolic blood pressure, serum glucose and free fatty acids, and some inflammatory biomarkers in healthy subjects [13]. A comprehensive obesity prevention report recommended that each country should promote food intake high in fruits and vegetables which are the main sources of antioxidants [14]. Currently a number of ongoing clinical studies are testing the effects of a number of substances including antioxidants on inflammation and metabolic and cardiovascular outcomes in obese patients [15].

We used a combination of antioxidant vitamins in our study because particular benefits from supplementing with vitamin C and E simultaneously derive from the interaction of these vitamins *in vivo*. Alpha-tocopherol is found mainly in the lipid compartment of cells and extracellular fluid, whereas ascorbic acid is located in the aqueous compartments. However, *in vitro* and, recently, *in vivo* studies point to an interaction such that there may be a mutual “sparing” effect [16]. Ascorbic acid appears to be able to regenerate the reduced, antioxidant form of α-tocopherol through redox cycling [17]. Although B-group vitamins reduced total plasma homocysteine concentrations in this study; they had no additive effects with antioxidants on markers of oxidative damage. This lack of difference may have been due to the lower dose of vitamins used, length of treatment, tissue inflammation, or differences in rate of absorption between subjects or due the small sample size of the study population. Homocysteine may promote oxidative stress through its detrimental effect on the vascular endothelium [6,18]. Therefore, homocysteine lowering with B-group vitamins may have an indirect antioxidant effect as a result of the decrease in vascular endothelial dysfunction.

Our study sample comes from a population known to have increased visceral obesity which is known to be associated with increased oxidative stress and inflammation [19]. This is because visceral fat secretes a number of factors involved in a range of metabolic and physiological processes, some of these factors having been implicated in the pathologies associated with obesity including diabetes, hypertension and CVD [3,4].

### Limitations and strength of the study

Our sample was small and almost one quarter of subjects refused to give a blood sample at 3 months follow up. Because the daily supplements dose we originally planned to use could only be contained in 3 separate capsules we ended up using a 1/3 of the daily dose (a capsule day) to help aid compliance. It is very likely that a larger dose of the supplement might have produced larger effects on oxidative damage and inflammation. The study has much strength including the random generation of the allocation sequence and allocation of supplements and also the placebo controlled design and the double-blind nature of intervention treatment and assessment.

## Conclusions

In conclusion, we have demonstrated in this study that supplementation with antioxidant and B-group vitamins improved antioxidant status and reduced tissue inflammation albeit in a small amount. If these results translate into clinical measurable benefit in obese diabetic patients, this simple treatment is likely to have considerable public health implications. Future larger studies with clinical endpoints are therefore needed to explore the potential therapeutic benefit of early antioxidant and B-group supplement and/or increased fruits and vegetables intake on evolution, progression and complications of obesity, diabetes and related cardiovascular diseases.

## Competing interest

The authors declare that they have no competing interests.

## Authors’ contribution

SG wrote the first draft, BA contributed to the discussion, MA & JY contributed to writing the manuscript and the discussion, HH and WI contributed to analysis of the data and the discussion, All authors read and approved the final manuscript.

## Funding

Faculty of Medicine & Health Sciences, United Arab Emirates University Project Grant.
